# In Vitro Anti-Inflammatory and Cytotoxic Effects of Aqueous Extracts from the Edible Sea Anemones *Anemonia sulcata* and *Actinia equina*

**DOI:** 10.3390/ijms18030653

**Published:** 2017-03-17

**Authors:** Tânia Costa Silva, Paula Branquinho de Andrade, Fátima Paiva-Martins, Patrícia Valentão, David Micael Pereira

**Affiliations:** 1 REQUIMTE/LAQV, Laboratório de Farmacognosia, Departamento de Química, Faculdade de Farmácia, Universidade do Porto, Rua de Jorge Viterbo Ferreira, no. 228, 4050-313 Porto, Portugal; atcsilva92@gmail.com (T.C.S.); pandrade@ff.up.pt (P.B.d.A.); valentao@ff.up.pt (P.V.); 2 REQUIMTE/LAQV, Departamento de Química e Bioquímica, Faculdade de Ciências, Universidade do Porto, Rua do Campo Alegre 1021/1055, 4169-007 Porto, Portugal; mpmartin@fc.up.pt

**Keywords:** inflammation, sea anemones, gastric cells, macrophages, homarine, cytotoxicity

## Abstract

Marine invertebrates have been attracting the attention of researchers for their application in nutrition, agriculture, and the pharmaceutical industry, among others. Concerning sea anemones (Cnidaria), little is known regarding their metabolic profiles and potential value as a source of pharmacologically-active agents. In this work, the chemical profiles of two species of sea anemones *Actinia equina* and *Anemonia sulcata*, were studied by high-performance liquid chromatography with diode-array detection (HPLC-DAD) and its impact upon immune and gastric cells was evaluated. In both species, the methylpyridinium alkaloid homarine was the major compound in aqueous extracts. The extracts were effective in reducing lipopolysaccharide (LPS)-induced levels of nitric oxide (NO) and intracellular reactive oxygen species (ROS) in a macrophage model of inflammation. Both the extracts and the alkaloid homarine were effective in inhibiting phospholipase A_2_ (PLA_2_), a pivotal enzyme in the initial steps of the inflammatory cascade. In order to mimic the oral consumption of these extracts; their effect upon human gastric cells was evaluated. While no caspase-9 activation was detected, the fact that the endoplasmic reticulum-resident caspase-4, and also caspase-3, were activated points to a non-classical mechanism of apoptosis in human gastric cells. This work provides new insights on the toxicity and biological potential of sea anemones increasingly present in human nutrition.

## 1. Introduction

Oceans cover more than 75% of the Earth’s surface and represent one of the most complex ecosystems, being an important resource for the discovery of novel molecules with distinct modes of action [1,2]. The interest in marine agents is growing and their biomedical potential has slowly gained prime significance among natural products’ research, attracting scientific and economic interests worldwide [1,2,3,4]. The chemical diversity of a wide variety of natural products from marine animals, have been providing therapeutic agents to treat various diseases, including those arising from acute or chronic inflammatory processes [2].

The phylum Cnidaria comprises over 11,000 species, including sea anemones [2]. In this work two sea anemone species were studied: *Actinia equina* (Linnaeus, 1767) and *Anemonia sulcata* (Pennant, 1777). These organisms are benthic as adults and lack the physical defense of a mineralized skeleton. Thus, they produce several secondary metabolites that often act as chemical defenses against predation, adverse conditions, fouling, or infection [5]. Nevertheless, little is described about their biological activity.

With the increasing consumption of novel foods there have been reports on the human consumption of several species of sea anemones as foodstuffs [6]. However, to the best of our knowledge, no previous work has addressed the composition of sea anemone extracts and their biological effects. In order to mimic the route of ingestion, we have used aqueous extracts of *A. equina* and *A. sulcata* with the aim of studying their in vitro anti-inflammatory effects in a murine macrophage cell line (RAW 264.7), as well as potential toxicity upon human gastric adenocarcinoma cells (AGS), with emphasis in the mechanism of action.

## 2. Results and Discussion

### 2.1. Chemical Profile of Aqueous Extracts

**Figure 1:**
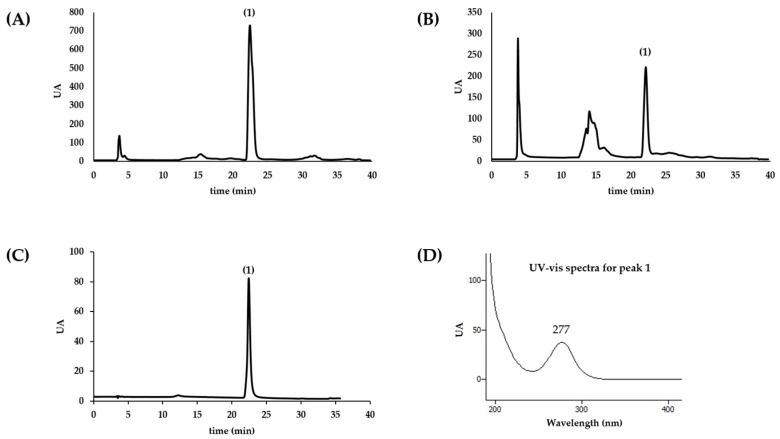
Chromatographic profile of the aqueous extract of sea anemones *A. equina* (**A**) and *A. sulcata* (**B**) and pure compound homarine (**C**) obtained by high-performance liquid chromatography with diode-array detection (HPLC-DAD) with detection at 280 nm and UV-VIS spectra of peak 1 (**D**). Peak 1 was identified as homarine.

The chromatographic profile of the aqueous extracts of the two species of sea anemones, *A. equina* and *A. sulcata*, is depicted in Figure 1 (A and B, respectively).

In both extracts the major peak corresponded to a molecule with the UV-VIS spectra presented in Figure 1, displaying an absorption maximum at 277.6 nm. In light of the chromatographic profile, spectra, information available in the literature and positive results from the alkaloid precipitation assays (results not shown), we hypothesized that the compound could correspond to the alkaloid homarine, which had been previously isolated from other species of sea anemones [7,8,9]. Given the fact that homarine was not commercially available at the time this work was conducted, we have synthesized this molecule. Homarine was identified as the major compound in both samples based on the analysis of the retention time, UV-VIS spectra, and co-injection of the pure compound with the samples. From a quantitative point of view, 1 mg/mL of aqueous extract of *A. equina* and *A. sulcata* contained homarine at the concentration of 0.49 and 0.23 µM, respectively. The isomer trigonelline, frequently detected in organic extracts of organisms along with homarine [10], was not detected under the same chromatographic conditions.

### 2.2. Biological Effects in Immune Cells

#### 2.2.1. Cytotoxic Effects of the Aqueous Extracts in RAW 264.7 Macrophages

**Figure 2:**
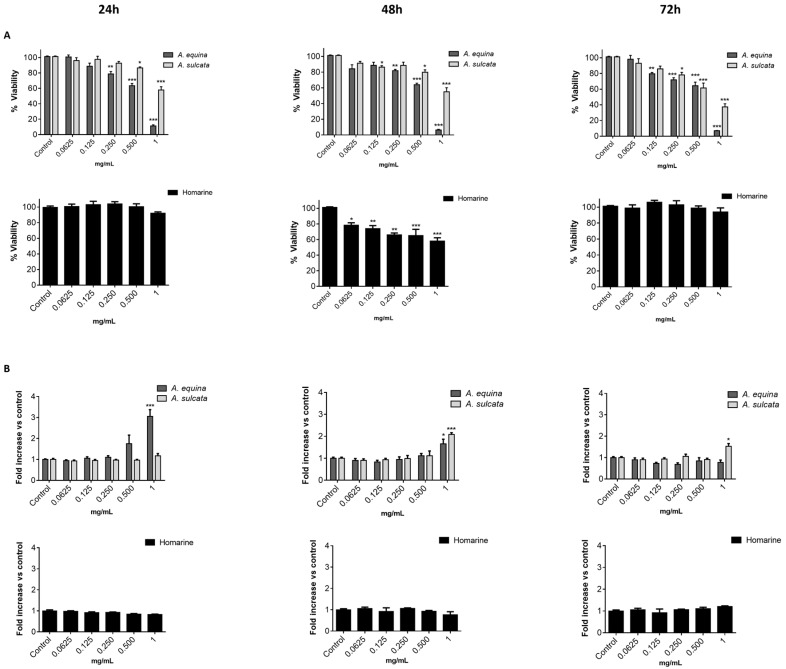
(**A**) Viability of RAW 264.7 macrophages treated with aqueous extracts of *A. equina* and *A. sulcata* and pure alkaloid homarine for 24, 48, and 72 h; (**B**) Evaluation of membrane integrity in RAW 264.7 macrophages. After 24, 48, and 72 h of exposure with the extracts and homarine, lactate dehydrogenase (LDH) was quantified. Statistical significance: * *p* < 0.05, ** *p* < 0.01, *** *p* < 0.001.

Prior to the assessment of their potential in vitro anti-inflammatory activity, the potential cytotoxicity of both aqueous extracts towards the macrophage cell line RAW 264.7 was evaluated by the 3-(4,5-dimethylthiazolyl-2)-2,5-diphenyltetrazolium bromide (MTT) reduction assay. RAW 264.7 cells were exposed to the extracts and homarine for 24, 48, and 72 h. At 24 h, a concentration-dependent toxic effect was found for the aqueous extract of *A. equina* (IC_50_ = 0.629 mg/mL) (Figure 2A) and, with the exception of concentrations of 0.0625 and 0.125 mg/mL, the remaining concentrations revealed significant differences when compared to the control. The aqueous extract of *A. sulcata* only revealed significant differences for concentrations equal or greater than 0.5 mg/mL. The highest concentration tested (1 mg/mL) decreased cell viability by 88.81% ± 2.69% with the aqueous extract of *A. equina* and by 42.07% ± 7.15% with the aqueous extract of *A. sulcata*. At the same concentration (1 mg/mL), homarine decreased cell viability by 7.86% ± 3.68% (Figure 2A). When comparing these results with those from higher incubation periods, it is possible to observe that the behavior of the extract from *A. equina* does not reveal major differences, while the extract of *A. sulcata* is increasingly more toxic with time, causing a reduction in cell viability around 62.63% ± 7.01% after 72 h at the highest concentration tested (1 mg/mL). Furthermore, statistical analysis revealed that with the course of time, *A. equina* and *A. sulcata* did not reveal significant differences (*F* value = 0.103 and *p* = 0.902 for *A. equina* and *F* value = 1.631 and *p* = 0.205 for *A. sulcata*). Homarine displays higher cytotoxicity at 48 h when compared to 24 and 72 h, the highest concentration tested (1 mg/mL) decreasing cell viability by 41.93% ± 7.35% (Figure 2A), as shown by the statistical differences found for 72 and 24 h (*p* < 0.0001 for both). The lower toxicity of homarine at 72 h could be the result of either metabolization of the extracts/homarine by cells or activation of defense mechanisms by cells.

Evaluation of membrane integrity was performed by monitoring LDH release in RAW 264.7 macrophages after 24, 48, and 72 h of treatment with the extracts or homarine (Figure 2B). At 24 h, while no effect was found for *A. sulcata* or homarine, the extracts of *A. equina* caused membrane disruption at the highest concentration tested (1 mg/mL). In light of these results, in subsequent assays, only non-necrotic concentrations (LDH negative) were used. Opposed to what was observed at 24 h, at 48 h both extracts induced significant increases in LDH release at the highest concentration tested (1 mg/mL). Nonetheless, at 72 h only the extract from *A. sulcata* resulted in increased LDH release. Homarine only showed significant differences in LDH release at 72 h, and only in the highest tested concentration (1 mg/mL).

#### 2.2.2. Involvement of Caspases 

**Figure 3:**
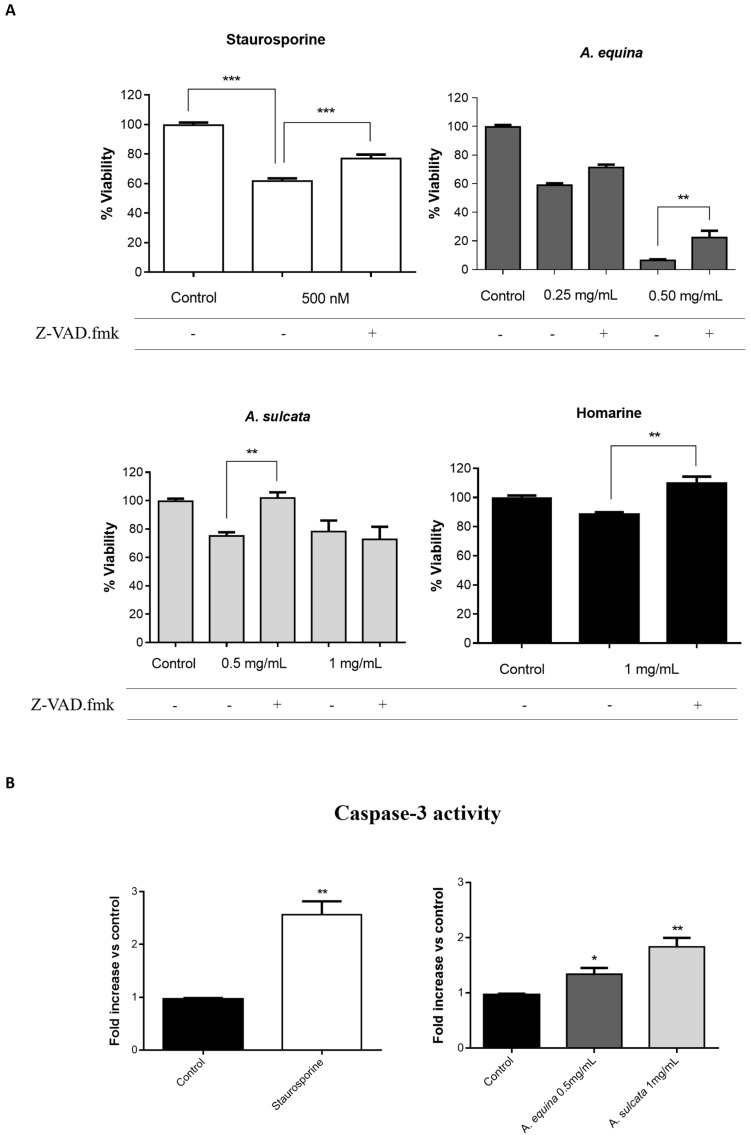
(**A**) Protective effect of the pan-caspase inhibitor Z-VAD.fmk in RAW 264.7 cells treated with aqueous extracts of sea anemones and homarine for 8 h; (**B**) Caspase-3 activity in RAW 264.7 cells treated with the aqueous extracts of *A. equina* and *A. sulcata* for 8 h. Staurosporine was used as a positive control for caspase activation and caspase inhibition. Results are presented as mean ± standard error of the mean of four independent experiments performed in triplicates. Statistical significance: * *p* < 0.05, ** *p* < 0.01, *** *p* < 0.001.

In order to evaluate the role of caspases in macrophage cell death elicited by the extracts and the major metabolite homarine, the pan-caspase inhibitor carbobenzoxy-valyl-alanyl-aspartyl-(*O*-methyl)-fluoromethylketone (Z-VAD.fmk) was used, after which the putative protective effect in cell viability was measured by the MTT reduction assay. As seen in Figure 3A, the toxic effect elicited by the extracts of *A. equina* and *A. sulcata*, as well as homarine, was prevented or ameliorated by pre-incubation with Z-VAD.fmk in some concentration. After the involvement of caspases was confirmed, we were interested in identifying the isoforms involved. As shown in Figure 3B, incubation of cells with the extracts of *A. sulcata* (1 mg/mL) and *A. equina* (0.5 mg/mL) resulted in increased activity of caspase-3. The activity of caspase-9 was also evaluated, however, no changes were noticed after incubation with both extracts or homarine (data not shown).

Taken together, the results from cell viability, membrane integrity, pharmacological inhibition of caspases and assessment of caspase-3/9 activity show that high concentrations of aqueous extracts of the sea anemones *A. sulcata* and *A. equina*, along with the major metabolite homarine, trigger a process of caspase-dependent cell death in macrophages.

#### 2.2.3. Aqueous Extracts of Sea Anemones Decrease Nitric Oxide (NO) and Reactive Oxygen Species (ROS) Levels in Lipopolysaccharide (LPS)-Stimulated Macrophages

Marine-derived molecules have been a prolific source of new anti-inflammatory drugs [11,12,13], reason for which we have conducted screening studies on the anti-inflammatory activity of the aqueous extracts of both sea anemones and their major metabolite, the alkaloid homarine.

Macrophages exposed to LPS develop an inflammatory phenotype as a consequence of the activation of Toll-like receptor 4 (TLR4). In this situation, macrophages present a number of biochemical traits, such as increased production of NO via inducible nitric oxide synthase (iNOS) [11] and increased levels of intracellular ROS.

**Figure 4:**
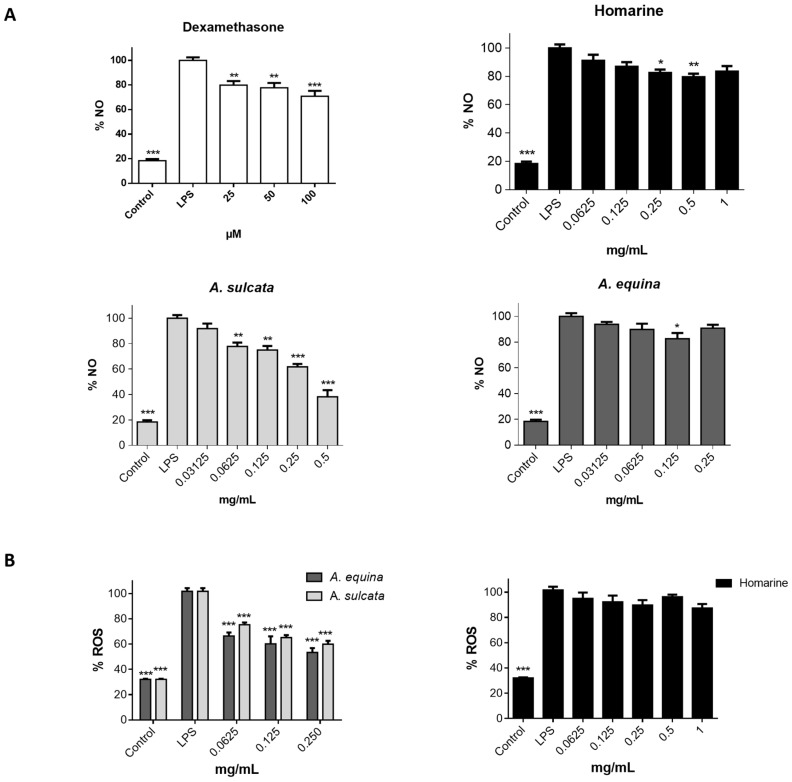
(**A**) Effect of both aqueous extracts, homarine and dexamethasone (positive control) on NO production by LPS-stimulated RAW 264.7 macrophages; (**B**) Effect of aqueous extracts and homarine in intracellular ROS levels of LPS-challenged cells, assessed by the fluorescent probe DCDHF-DA. Results are presented as mean ± standard error of the mean of six independent experiments performed in triplicate. Statistical significance: * *p* < 0.05, ** *p* < 0.01, *** *p* < 0.001.

Cells were pre-incubated with both extracts, homarine, or the reference anti-inflammatory drug dexamethasone for 2 h and then exposed to LPS for 22 h. As shown in Figure 4A, at non-toxic concentrations the aqueous extract of *A. sulcata* was able to decrease NO levels in a dose-dependent way (IC_50_ = 0.374 mg/mL). At the highest concentration tested (0.5 mg/mL), *A. sulcata* decreased NO levels by 61.85% ± 11.74%. Concerning *A. equina*, only the concentration 0.125 mg/mL was able to reduce NO levels. Homarine, the major metabolite found in both extracts, was able to significantly reduce NO levels at 0.25 and 0.5 mg/mL. In addition, statistical analysis shows that NO reduction by *A. sulcata* is significantly different (*F* value = 14.753) than that of *A. equina* (*p* < 0.0001) and homarine (*p* = 0.0002).

ROS, such as superoxide anion radicals, are able to combine with NO in order to form reactive nitrogen species, such as peroxynitrite, which induce nitrosative stress that further increases the pro-inflammatory status [14]. Given the fact that increased production of ROS is one of the biochemical events following TLR4 activation by LPS, the ability of the sea anemone extracts to counter this augmented oxidative status was evaluated. Cells were pre-incubated with both extracts, or homarine, for 2 h, and then exposed to LPS for 22 h. After this, intracellular ROS were evaluated. The results show that both extracts were able to decrease ROS in LPS-challenged cells by approximately 30% in concentrations as low as 0.0625 mg/mL (Figure 4B. In the concentration range used, homarine failed to significantly decrease ROS generation. Furthermore, statistical analysis (*F* value = 17.607) shows that both extracts, *A. sulcata* and *A. equina*, display similar capacity to reduce intracellular ROS (*p* = 0.107). 

### 2.3. Sea Anemone Extracts Inhibit Phospholipase A_2_ (PLA_2_)

In light of the positive results in the NO screening assay for anti-inflammatory activity, we were interested in studying the possible target responsible for this effect. The inflammatory cascade is a complex succession of events that result in the production of pro-inflammatory mediators. The first step, however, involves the PLA_2_ family of enzymes, which recognize the sn-2 acyl bond of phospholipids and catalytically hydrolyze the bond releasing, arachidonic acid (AA) and lysophospholipids [15]. After this, AA can be the substrate of cyclooxygenases and lipoxygenase (LOX), being converted into active compounds named eicosanoids [16], which have been associated with the pathogenesis of several human inflammatory diseases [13]. In order to elucidate possible mechanisms of anti-inflammatory activity, the ability of the extracts to inhibit the first steps of the inflammatory cascade was evaluated, in particular PLA_2_.

**Figure 5:**
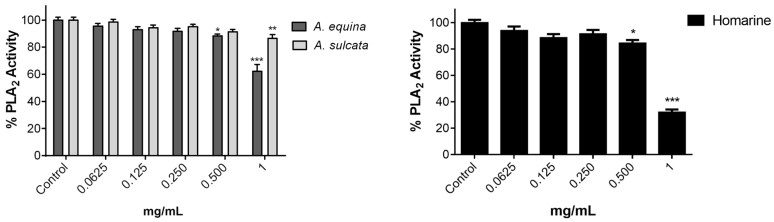
Effect of both sea anemones aqueous extracts and homarine on PLA_2_ activity in a non-cellular system. Results are presented as mean ± standard error of the mean of four independent experiments performed in triplicates. Statistical significance: * *p* < 0.05, ** *p* < 0.01, *** *p* < 0.001.

Both extracts revealed significant inhibition of the enzyme, the extract from *A. equina* being more potent (Figure 5). The aqueous extract of *A. sulcata* only exhibited significant inhibition at 1 mg/mL, while *A. equina* displayed inhibition in the 0.5 and 1 mg/mL, reaching 37.75 ± 8.43 of inhibition at the highest concentration. The same concentration range was found for homarine, which inhibited PLA_2_ by over 65% at the two highest concentration tested. 

### 2.4. Biological Effects of Sea Anemone Extracts in Human Gastric Cells

#### 2.4.1. Sea Anemone Extracts Display Cytotoxicity

**Figure 6:**
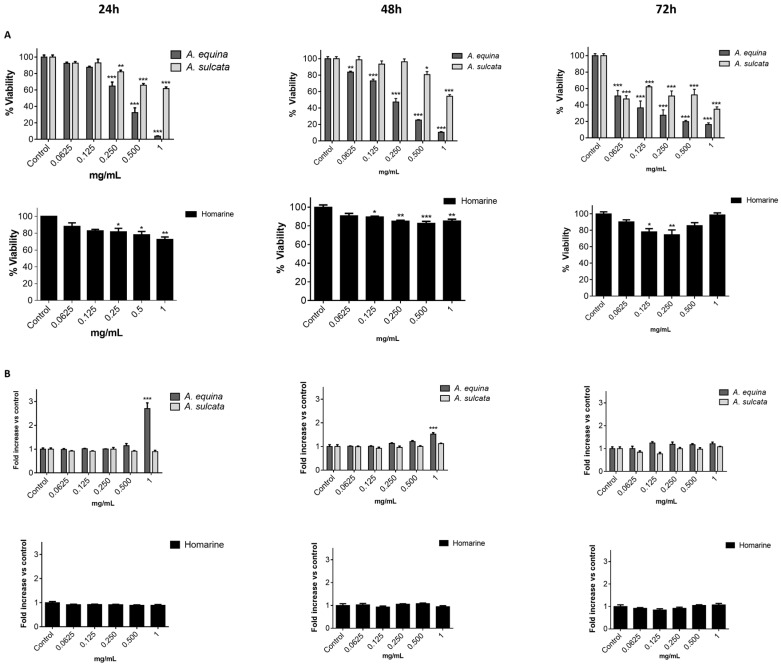
(**A**) Viability of AGS treated with aqueous extracts of *A. equina* and *A. sulcata* or homarine for 24, 48, and 72 h; (**B**) Evaluation of membrane integrity in AGS cells. After 24, 48, or 72 h of exposure with the extracts and homarine, LDH activity was quantified. Results are presented as mean ± standard error of the mean of three independent experiments performed in triplicates. Statistical significance: * *p* < 0.05, ** *p* < 0.01, *** *p* < 0.001.

In order to mimic the ingestion route of aqueous extracts of anemones, their impact upon human gastric cell viability was evaluated by the MTT assay. AGS cells were exposed to the extracts or homarine for 24, 48, and 72 h. As depicted in Figure 6A, after 24 h of incubation the cytotoxic effect of both extracts was concentration-dependent in the same concentration range, the aqueous extract of *A. equina* (IC_50_ = 0.365 mg/mL) being more cytotoxic. The highest concentration of *A. equina* (1 mg/mL) decreased cell viability by 96.08% ± 0.66%, while in the case of *A. sulcata* decreased cell viability by 38.32% ± 4.06%.

In order to correlate the cytotoxic effects of the aqueous extracts with their chemical profile, the effect of the major compound, homarine, was also evaluated. As shown in Figure 6A, homarine caused significant concentration-dependent toxic effects and decreased cell viability by 27.57% ± 5.42% at the highest concentration tested (1 mg/mL, Figure 6A). In the case of *A. sulcata*, the toxicity was largely coincident with that of homarine. When comparing the influence of the incubation period in the toxicity displayed, results show that the course of time does not significantly impact the effect of the extracts or homarine on cell viability. 

The effect of both the extracts and homarine on the membrane integrity of AGS cells was evaluated after 24, 48, and 72 h of incubation by the LDH assay (Figure 6B). At 24 h, the extract of *A. sulcata* had no impact in the integrity of the membrane, as shown by the absence of LDH in the extracellular environment, while loss of membrane integrity was found at the highest concentration of *A. equina* (1 mg/mL). For this reason, this concentration was not used in subsequent assays. Differently from what was verified at 24 h of incubation, at 48 h the highest concentrations of both extracts displayed significant increases in LDH release. However, at 72 h none of extracts showed a significant increase in LDH release, which could be the result of degradation of the LDH enzyme through time. Homarine did not reveal significant differences in LDH release in the highest tested concentration (1 mg/mL) at 72 h.

#### 2.4.2. Impact on Cell Morphology

**Figure 7:**
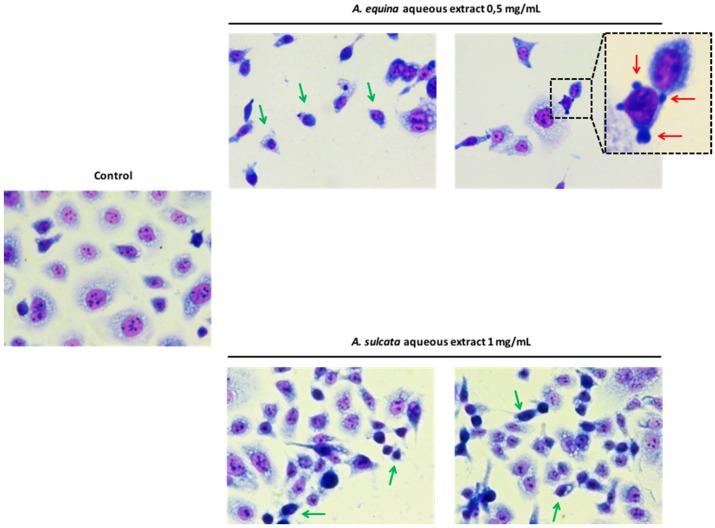
Morphological assessment of AGS cells (control vs. treatments, 24 h of incubation) by Giemsa staining. Green arrow: condensed chromatin; red arrow: apoptotic bodies. Magnification 400×.

In order to better understand the mechanism of cytotoxicity displayed by the extracts and their constituents, the effect on cell morphology was assessed after 24 h of incubation. As it can be seen in Figure 7, incubation with the extract of *A. equina* (0.5 mg/mL) and *A. sulcata* (1 mg/mL) resulted in chromatin condensation (Figure 7, green arrows) and decreased cell size when compared to control cells. Furthermore, in cells treated with 0.5 mg/mL of *A. equina* extract, structures compatible with apoptotic bodies were found (Figure 7, red arrows), suggesting the occurrence of a type of programmed cell death [17]. 

#### 2.4.3. Toxicity of Sea Anemone Extracts Involves Activation of Caspases

**Figure 8:**
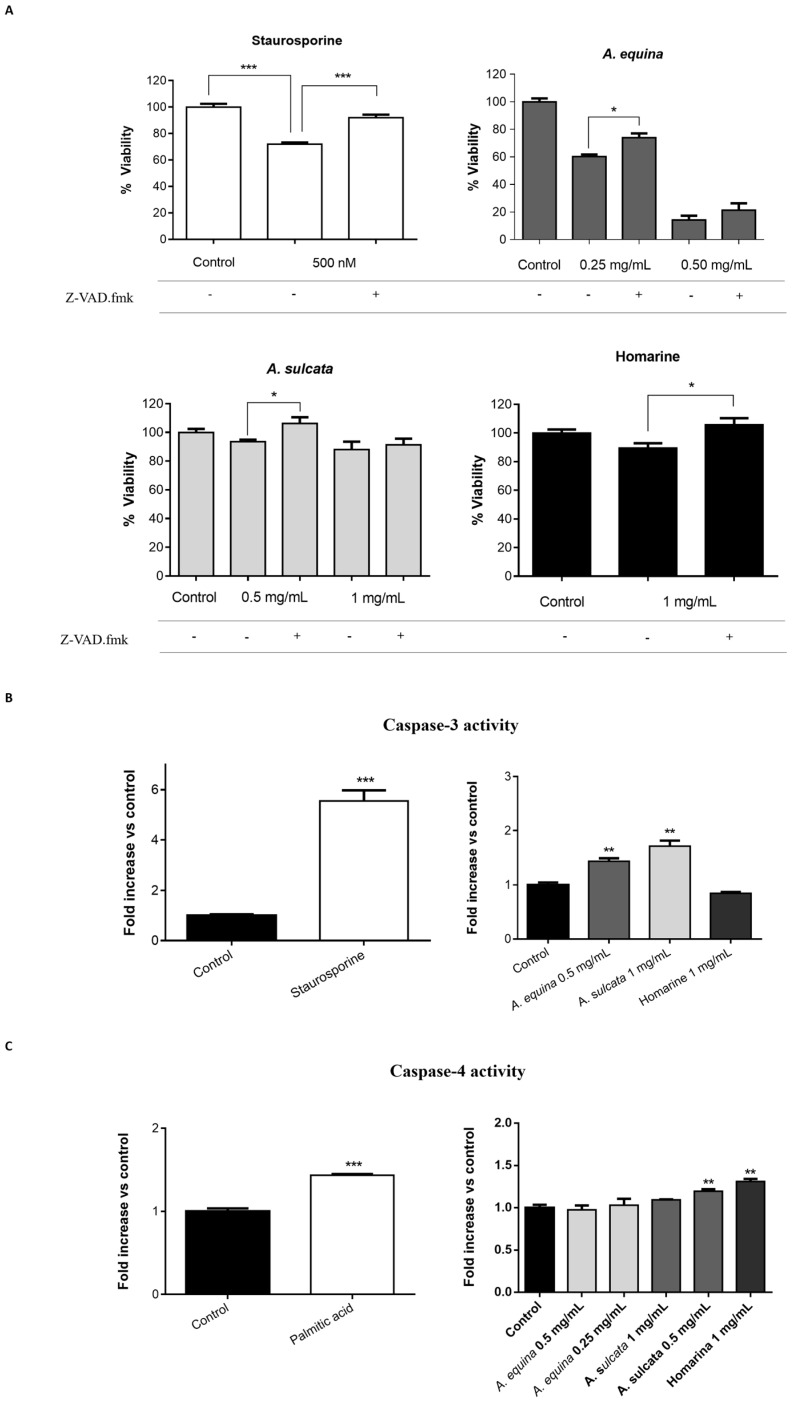
(**A**) Effect of Z-VAD.fmk in AGS cell death caused by extracts and homarine, evidencing that incubation with the pan-caspase inhibitor rescues cell from toxicity; (**B**) Caspase-3 activity in AGS cells treated with aqueous extracts and homarine. At the concentrations indicated, the extracts caused an increase in caspase-3 activity. Staurosporine (500 nM) was used as a positive control for caspase activity; (**C**) Caspase-4 activity in AGS cells treated with the aqueous extracts and homarine. At the concentrations indicated, the extracts of *A. sulcata* and homarine caused an increase in caspase-4 activity. Palmitic acid (1 mM) was used as a positive control. Results are presented as mean ± standard error of the mean of four independent experiments performed in triplicates. Statistical significance: * *p* < 0.05, ** *p* < 0.01, *** *p* < 0.001.

In light of the results obtained in cell viability and morphological assessment, we hypothesized that caspases could be involved in the toxicity triggered by the extracts. To confirm this, the putative protective effect of the pan-caspase inhibitor Z-VAD.fmk upon cell viability [18] was evaluated by the MTT reduction assay (Figure 8A). In order to study cellular events upstream of the toxicity found at 24, 8 h of incubation was used. Results show that Z-VAD.fmk was able to reduce the toxicity of homarine (1 mg/mL), *A. equina* (0.25 mg/mL), and *A. sulcata* (0.5 mg/mL) (Figure 8A). For this reason, in subsequent experiments the activity of several caspase isoforms was evaluated. As shown in Figure 8B, incubation of cells with aqueous extracts of *A. equina* (0.5 mg/mL) and *A. sulcata* (1 mg/mL) resulted in increased caspase-3 activity. In the case of homarine, while the pan-caspase inhibitor Z-VAD.fmk was able to reduce the toxicity, no caspase-3 activation was observed in the tested period, which could be a result of different kinetics of caspase activation with the pure alkaloid. Both extracts exhibit similar potential to significantly increase caspase-3 activity, when compared to the control group (*F* value = 0.753 and *p* = 0.403).

Given the fact that caspase-3 is an effector caspase, we were interested in evaluating the possible initiator caspase that precedes its activation. In the classical intrinsic pathway, activation of the initiator caspase-9 takes place, subsequently activating caspase-3 [19]. This was not the case here, as neither the extracts, nor the alkaloid homarine were able to activate caspase-9 in the conditions assayed (data not shown). In order to investigate the possible upstream target leading to caspase-3 activation, we have tested the ability of the extracts and homarine to activate caspase-4, an endoplasmic reticulum (ER)-resident caspase that has been previously associated with caspase-3 activation in cases of ER-dependent apoptosis [19]. Results show that caspase-4 activity was increased after incubation with the aqueous extracts of *A. sulcata* (0.5 mg/mL) and homarine (1 mg/mL) (Figure 8C). For this reason, we can conclude that the aqueous extracts of sea anemone *A. sulcata* and its major metabolite homarine are cytotoxic to human gastric cancer cells by triggering a non-classical type of cell death that is mediated by caspase-4 and -3, with the putative involvement of the ER.

## 3. Materials and Methods 

### 3.1. Chemicals and Standards

LPS from *Escherichia coli*, sulfanilamide, dichlorodihydrofluorescein diacetate (DCDHF-DA), MTT, DPX mountant, sodium pyruvate, β-nicotinamide adenine dinucleotide reduced form (NADH), Triton X-100, *N*-(1-naphthyl)ethylenediamine dihydrochloride, Giemsa dye, sodium deoxycholate, trizma hydrochloride, dimethyl sulfoxide (DMSO), LOX from *Glycine max* (L.) Merr. (Type V-S; EC 1.13.11.12), PLA_2_ (EC.3.1.1.4, from honey bee venom (*Apis mellifera*), magnesium chloride (MgCl_2_), 1,2-dilinoleoyl-sn-glycero-3-phosphocholine, Trypan blue, iodomethane, propylene carbonate, picolinic acid, propano-2-ol, 3-[(3-cholamidopropyl)dimethylammonio]-1-propanesulfonate hydrate (CHAPS), sucrose, 1,4-dithiothreitol (DTT), 2-[4-(2-hydroxyethyl)piperazin-1-yl]ethanesulfonic acid (HEPES), 2,2′,2″,2′′′-(ethane-1,2-diyldinitrilo)tetraacetic acid (EDTA), palmitic acid, dexamethasone and phorbol 12-myristate 13-acetate (PMA) were from Sigma-Aldrich (St. Louis, MO, USA). Dulbecco’s Modified Eagle Medium (DMEM), fetal bovine serum (FBS), Hank’s balanced salt solution (HBSS), 0.05% Trypsin-EDTA (1X) and penicillin-streptomycin solution (penicillin 5000 units/mL and streptomycin 5000 µg/mL) were purchased from GIBCO^®^ by life technologies™, Invitrogen (Grand Island, NY, USA). Caspase-3/-7 luminescent assay kit was from Promega Corporation. Staurosporine and Z-VAD.fmk were obtained by Santa Cruz Biotechnology (Dallas, Texas, USA). Acetonitrile, potassium dihydrogen phosphate and methanol were purchased from Merck (Darmstadt, Germany). Hydrochloric acid was from VWR International, LLC. Ammonia, phosphoric acid and ethyl ether were from Panreac (Barcelona, Spain). Ac-Leu-Glu-His-Asp-7-Amino-4-trifluoromethylcoumarin (caspase-9 substrate) was purchased from CPC Scientific (Sunnyvale, CA, USA). Z-Leu-Glu-Val-Asp-AFC (caspase-4 substrate) was obtained from Innovagen (Lund, Sweeden). Ethylene glycol-bis(2-aminoethylether)-*N*,*N*,*N*′,*N*′-tetraacetic acid was from AMRESCO (Solon, OH, USA). 

### 3.2. Sampling and Extract Preparation

Individuals of *A. sulcata* and *A. equina* were collected in Praia da Luz (Porto, Portugal) in July 2014. After collection, samples were placed on ice and immediately transported to the laboratory, washed with a saline solution to remove contaminants, frozen and lyophilized (Lyophilizer Lacone Freezone 4,5—Kansas City, MO, USA). Afterwards, the samples were pulverized and 5 g were sifted (910 µm). After this procedure 100 mL of extractor solvent (water) were added. The extraction occurred in 30 min under stirring (200 rpm) and at room temperature. Both extracts were centrifuged (ROTOFIX 32A, Hettich, Tuttlingen, Germany) at 400 rpm for 10 min and then filtered under reduced pressure. Lastly, aqueous extracts were frozen at −20 °C, lyophilized for seven days, and then stored in a desiccator until analysis.

### 3.3. Metabolic Profile Analysis

The chromatographic profile of aqueous extract of sea anemones was established by HPLC-DAD (Gilson 811C Dynamic Mixer, Gilson HPLC Dynamic Mixer). Compounds present in the extracts were separated with reverse phase column (Waters Spherisorb, C18, 5 µm ODS2, 250 × 4.6 mm Analytical Cartridge, Part NO. PSS839540, Dublin, Ireland), at room temperature and using a gradient elution method. The mobile phase consisted of two solvents: water (A) and acetonitrile (B). Elution started with 100% B and a gradient was used to obtain 85% A at 15 min, followed by 30% A at 35 min. The injection volume was 20 µL and the flow rate 0.9 mL/min. The spectral data were collected in the range of 190–700 nm and elution was monitored at 280, 320, and 350 nm. The data were processed using the software Clarity (Europa Science Ltd., Cambridge, UK). Homarine quantification was achieved by the interpolation of the absorbance recorded at 280 nm in the chromatograms relative to a calibration curve constructed with the authentic standard analyzed under the same conditions. At least three independent analysis were conducted.

### 3.4. Screening for Alkaloids

Screening for alkaloids was performed in order to assess the possible presence of these in both extracts [20]. For alkaloid extraction, extracts (1 g) were weighed, heated with 10 mL of 10% hydrochloric acid and then filtered. Ammonia solution (1:1) was added to alkalinize the solution and alkaloids were extracted with ethyl ether. The ether phase was separated and 10 mL of 10% HCl solution were added. Finally, the solution was divided in 4 test tubes, three of them receiving either the Dragendorff, Mayer, or Bertrand’s reagent, the fourth one serving as control. Formation of precipitates with all reagents were assessed.

### 3.5. Synthesis of Homarine

As there was no homarine commercially available at the time this study was conducted; as such, we have synthesized this molecule: 1.5 g iodomethane was added to a well-stirred suspension of 1.0 g picolinic acid (0.81 mmol) in 20 mL propylene carbonate. After two days, 100 mL ether were added and the resulting yellow solid was recrystallized in methanol/ether. Identity of the molecule was confirmed by ^1^H-NMR (400 MHz, MeOD) δ ppm: 4.55 (3H, s), 8.11 (1H, td, *J* = 7 Hz, *J* = 1 Hz), 8.36 (1H, dd, *J* = 8 Hz, *J* = 1 Hz), 8.65 (1H, td, *J* = 8 Hz, *J* = 1 Hz), 8.96 (1H, dd, *J* = 7 Hz, *J* = 1 Hz); ^13^C-NMR (101 MHz, MeOD) δ ppm: 49.00, 129.53, 129.59, 147.69, 148.58, 150.78, 163.73.

### 3.6. Cell Culture

Murine macrophage-like cell line, RAW 264.7, was from the American Type Culture Collection (LGC Standards S.L.U., Barcelona, Spain) and AGS cells were from Sigma-Aldrich. Cells were maintained in DMEM supplemented with 10% heat-inactivated FBS and 1% penicillin/streptomycin, and grown in an incubator at 37 °C, in a humidified atmosphere of 5% CO_2_. AGS cells were trypsinized and subjected to centrifugation at 1300 rpm for 5 min and then suspended in flasks. RAW cells were harvested by scraping and then suspended in flasks for growth.

### 3.7. MTT

Cell viability was evaluated by the MTT reduction assay [21]. Cells were cultured in 96-well plates (15,000 cells/well for AGS and 25,000 cells/well for RAW) and allowed to attach for 24 h. After incubation with extracts or molecules (0.0625–1 mg/mL) for 24, 48 and 72 h, MTT (0.5 mg/mL final concentration) was added to each well and the plate was incubated for 90 min at 37 °C. Formazan crystals were dissolved by addition of a DMSO:isopropanol mixture (3:1) and then quantified spectrophotometrically at 560 nm in microplate reader (Multiskan ASCENT, Haverhill, MA, USA). The results of cell viability correspond to the mean ± standard error of at least three independent experiments performed in triplicate and are expressed as the percentage of the untreated control cells. 

### 3.8. Morphological Studies

AGS cells were cultured at a density of 50,000 cells/well in coverslips placed in 24-well plates, as previously described [22], with some modifications. Briefly, after incubation with different concentrations of the aqueous extracts (0.5 mg/mL of *A. equina* and 1 mg/mL of *A. sulcata*) for 24 h, cells were washed twice with HBSS and fixed in coverslips with cold methanol, at 4 °C for 30 min. Giemsa dye (1:10) was then added and kept for 30 min at room temperature, after which cells were repeatedly washed with water and then mounted in DPX.

### 3.9. Membrane Integrity

In order to evaluate the membrane integrity, the activity extracellular LDH was measured as previously described [12]. Briefly, cells were cultured in 96-well plates as described for the MTT assay and allowed to attach for 24 h at 37 °C. After this, cells were incubated with extracts or the molecule (0.0625–1 mg/mL) for 24, 48, and 72 h. LDH released into the culture media after 24, 48, and 72 h in culture media supernatant was evaluated by monitoring the decrease of NADH (252.84 µM) during the conversion of pyruvate (14.993 mM) to lactate, at 340 nm in microplate reader (Multiskan ASCENT, Haverhill, MA, USA). Triton X-100 1% was used as positive control for cell lysis (30 min). All of the results correspond to the fold-increase of absorbance in treated versus untreated cells. 

### 3.10. Inhibition of PLA_2_

Inhibition of PLA_2_ assay was measured as previously described [15] with some modifications. Phospholipase A_2_ was solubilized in water and used at a final concentration of 0.25 µg/mL in 3 mM deoxycholate dissolved in 50 mM Tris-HCl buffer (pH 8.5). The solution of phosphatidylcholine substrate was prepared by drying aliquots of a dilinoleoyl phosphatidylcholine stock solution in chloroform under a stream of N_2_; the film obtained was rapidly dispersed, up to a final concentration of 1.3 mM, in 10 mM deoxycholate dissolved in 50 mM Tris buffer, pH 8.5. In the assay, the substrate was used in a final concentration of 65 μM in 50 mM Tris-HCl buffer (pH 8.5). Type V lipoxygenase from *Glycine max* (soybean) LOX was used as a co-enzyme in a final concentration of 0.23 µg/mL in 3 mM deoxycholate dissolved in 50 mM Tris-HCl buffer, pH 8.5. The extracts were dissolved in the same buffer. Both enzymes were mixed with 50 µL of the extracts or molecule (0.0625–1 mg/mL) and the reaction started by the adding of PLA_2_ substrate. The linoleic acid released by PLA_2_ is oxidized by 5-LOX and then phospholipase activity was assessed by monitoring the formation of conjugated dienes of the hydroperoxide product at 234 nm in a microplate reader (Multiskan ASCENT, Haverhill, MA, USA).

Controls without either PLA_2_, 5-LOX or substrate were always carried out. All assays were performed at 37 °C and each measurement was repeated three times. Values represent the means ± standard error of the mean and are expressed as percentage of the control with enzymes and substrate. 

### 3.11. Evaluation of NO Levels

RAW 264.7 cells were cultured in 96-well plates (35,000 cells/well) for 24 h and then pre-treated with different concentrations of aqueous extract of each sea anemone, homarine, and dexamethasone, for 2 h. Afterwards, LPS was added (final concentration 1 µg/mL) and the plates were incubated for 22 h at 37 °C, in a humidified atmosphere of 5% CO_2_. The nitrite resulting from the conversion of NO in the culture medium was quantified by mixing 75 µL of culture media with an equal volume of Griess reagent (1% sulfanilamide and 0.1% naphthylethylenediamine dihydrochloride in 2% H_3_PO_4_) and incubated for 10 min in the dark, after which absorbance was read at 540 nm in a microplate reader (Multiskan ASCENT, Haverhill, MA, USA). The results correspond to the mean ± standard error of the mean of five independent experiments performed in triplicate and are expressed as percentage of NO in cells exposed to LPS (positive control for NO production). 

### 3.12. Intracellular ROS Levels

Cells were seeded in 96-well black plates (15,000 cells/well for AGS and 25,000 cells/well for RAW 264.7) according to the above-mentioned conditions for the MTT assay. After this, cells were incubated with extracts (0.0625–0.25 mg/mL) or homarine (0.0625–1 mg/mL) for 24 h. Cells were washed with HBSS for 30 min before the end of the incubation period, followed by incubation with a solution of DCDHF-DA (25 µM in HBSS) for 45 min at 37 °C. The quantification of intracellular ROS was performed using fluorescence microplate reader (Cytation™ 3, BioTek, Winooski, VT, USA) (Excitation: 490 nm excitation; Emission: 520 nm).

### 3.13. Caspase Inhibition Assays

AGS and RAW 264.7 cells were cultured in 96-well plates as described for the MTT assay and allowed to attach for 24 h. After incubation, cells were pre-incubated with Z-VAD.fmk (AGS and RAW 264.7 cells) (50 µM) for 1 h and then extracts (0.25 and 0.5 mg/mL of *A. equina* and 0.5 and 1 mg/mL of *A. sulcata*) or homarine (1 mg/mL) were added and incubated for 8 h at 37 °C. After this, MTT assay was conducted.

### 3.14. Caspases Activity Assays

#### 3.14.1. Caspase-3

Activity of caspase-3 was monitored using the Caspase-Glo^®^ 3/7 kit Assay. After incubation in 96-well white plates of RAW 264.7 and AGS cells, as previously described for the MTT assay, cells were treated with the extracts (0.5 mg/mL of *A. equina* and 1 mg/mL of *A. sulcata*) or homarine (1 mg/mL) and incubated for 8 h at 37 °C. After this Caspase-Glo^®^ 3/7 buffer and Caspase-Glo^®^ 3/7 substrate were added to supernatant of the cells in the same quantity during 35 min at 22 °C and the luminescent signal was measured using a microplate reader (Cytation™ 3, BioTek, Winooski, VT, USA). In both cell lines, caspase-3 is the effector caspase and, thus, results from the Caspase-Glo^®^ 3/7 kit Assay can be attributed to caspase-3. Staurosporine was used as a positive control. 

#### 3.14.2. Caspase-4

For the assessment of caspase-4 activity, AGS cells were cultured in 96-well black plates, as previously described for the MTT assay, and allowed to attach for 24 h. After this, cells were incubated with extracts (0.25 and 0.5 mg/mL for *A. equina* and 0.5 and 1 mg/mL for *A. sulcata*) or homarine (1 mg/mL) for 8 h at 37 °C and then the supernatant was removed and substrate of caspase-4 (50 µM) was incubated for 150 min at 37 °C. Fluorescence was determined in a microplate reader (Cytation™ 3, BioTek, Winooski, VT, USA) (Excitation: 400 nm; Emission: 505 nm). Palmitic acid (1 mM) was used as a positive control for caspase-4 activation. 

#### 3.14.3. Caspase-9

AGS and RAW 264.7 cells were cultured in 12-wells plates (75,000 cells/well and 200,000 cells/well, respectively), as described for the MTT assay, and allowed to attach for 24 h. After this, cells were pre-incubated with extracts (0.25 and 0.5 mg/mL for *A. equina* and 0.5 and 1 mg/mL for *A. sulcata*) or homarine (1 mg/mL) for 8 h at 37 °C, after which the culture medium was removed and cells were washed with HBSS. RAW 264.7 cells were harvested by scraping and AGS cells were trypsinized and subjected to centrifugation at 1300 rpm for 3 min. Afterwards, lysis buffer (25 mM HEPES, 5 mM EDTA, 1 mM EGTA, 5 mM MgCl_2_, and 5 mM DTT, pH 7.4) was added for 5 min, with subsequent centrifugation at 14,500 rpm for 15 min, at 4 °C. The supernatants were removed and assayed for protein content by the Bradford method [23]. In order to measure caspase activity, aliquots of cell extracts containing 50 µg of protein were added to a reaction buffer (25 mM HEPES, 0.1% CHAPS, 10% sucrose, and 5 mM DTT pH 7.4). The reaction was initiated after addition of the substrate for caspase-9 (50 µM) and fluorescence was determined after 90 min for RAW 264.7 cells and 120 min for AGS cells at 37 °C, in a microplate reader (Cytation™ 3, BioTek, Winooski, VT, USA) (Excitation: 405 nm; Emission: 535 nm).

### 3.15. Statistical Analysis

Statistical analysis was performed using KaleidaGraph 4.0 Synergy Software Inc (Reading, PA, USA). One-way ANOVA, followed by a Scheffe multiple range test, was used to determine the statistical significance in comparison to cells treated and untreated. *F* values of all statistical analysis are shown in the Tables S1–S3 of Supplemental Information. Data are expressed as the mean ± standard error of the mean. In all cases, values of *p* ≤ 0.05 were considered statistically significant.

## 4. Conclusions

This work constitutes the first study addressing the chemical composition and biological effects of aqueous extracts from the sea anemones *A. equina* and *A. sulcata*. Metabolite profiling of these species showed that, in both cases, the alkaloid homarine was the major metabolite. 

*A. equina* was more toxic that *A. sulcata* for both gastric and macrophage cells. Both extracts revealed to exert their toxic effects by activation of caspase-3. Furthermore, both extracts were effective in preventing LPS-induced increase of NO, in addition to being able to significantly lower the ROS levels produced by macrophages when displaying an inflammatory phenotype. In addition, *A. equina* showed mild ability to inhibit PLA_2_. In several of the parameters studied, such as NO production and PLA_2_ inhibition, the major metabolite homarine displayed significant activity that could be responsible for the activity of the extracts, however we cannot rule of the possible contribution of other molecules present.

This is the first report on the toxicity of aqueous extracts of this species. In light of the recent interest of sea anemones as gourmet ingredients, our results on the toxicity of these organisms justify further studies to further assess the safety of sea anemones for human consumption. In addition, we show that at sub-toxic concentrations the aqueous extracts display mild anti-inflammatory activity, thus, raising the interest in the exploitation of this material as a source of bioactive molecules.

## Supplementary Materials

Supplementary materials can be found at www.mdpi.com/1422-0067/18/3/653/s1.
