# Supplementary material for: In Vitro Anti-Inflammatory and Cytotoxic Effects of Aqueous Extracts from the Edible Sea Anemones Anemonia sulcata and Actinia equina
Source: Int J Mol Sci. 2017 Mar 17;18(3):653. doi: 10.3390/ijms18030653 (PMC5372665; doi:10.3390/ijms18030653)
Supplement: Supplementary file 1 [file ijms-18-00653-s001.pdf]

Supplementary Materials: In Vitro Anti-Inflammatory and Cytotoxic Effects of Aqueous Extracts from the Edible Sea Anemones *Anemonia sulcata* and *Actinia equina*

Tânia Costa Silva, Paula Branquinho de Andrade, Fátima Paiva-Martins, Patrícia Valentão and David Micael Pereira

Table S1. *F* values for statistical analysis of MTT and LDH assay for AGS and RAW 264.7 cells, and NO and ROS inhibition assay for RAW 264.7 cells.

Cells	Assay	Time (h)	Extract/Compound	*F* value
RAW 264.7	MTT	24	*A. equina*	171.942
			A. sulcata	31.705
			Homarine	2.483
		48	*A. equina*	140.485
			A. sulcata	24.584
			Homarine	16.139
		72	*A. equina*	146.282
			A. sulcata	38.531
			Homarine	1.416
	LDH	24	*A. equina*	18.234
			A. sulcata	2.349
			Homarine	4.985
		48	*A. equina*	6.562
			A. sulcata	14.952
			Homarine	1.375
		72	*A. equina*	1.506
			A. sulcata	8.812
			Homarine	1.918
	NO production	24	*A. equina*	107.556
			A. sulcata	75.102
			Homarine	82.209
	ROS production	24	*A. equina*	59.409
			A. sulcata	167.781
			Homarine	60.309
AGS	MTT	24	*A. equina*	138.428
			A. sulcata	40.272
			Homarine	8.907
		48	*A. equina*	227.010
			A. sulcata	27.869
			Homarine	10.997
		72	*A. equina*	34.461
			A. sulcata	33.851
			Homarine	8.260
	LDH	24	*A. equina*	38.653
			A. sulcata	1.218
			Homarine	2.065
		48	*A. equina*	20.553

		A. sulcata	1.228
		Homarine	0.981
	72	*A. equina*	1.694
		A. sulcata	4.022
		Homarine	1.927

Table S2. *F* values for statistical analysis of caspases assay for both cells.

**Cells**	**Assay**	**Time (h)**	**Extract/Compound**	**Concentration (mg/mL)**	***F* value**
**RAW 264.7**	Pan caspase inhibitor Z-VAD.fmk	8	*A. equina*	0.25	3.131
				0.5	27.221
			A. sulcata	0.5	0.199
				1	31.420
			Homarine	1	18.607
	Caspase-3 activation	8	*A. equina*	0.5	10.905
			A. sulcata	1	27.421
**AGS**	Pan caspase inhibitor Z-VAD.fmk	8	*A. equina*	0.25	16.268
				0.5	1.461
			A. sulcata	0.5	7.740
				1	0.215
			Homarine	1	8.043
	Caspase-3 activation	8	*A. equina*	0.5	34.273
			A. sulcata	1	31.065
			Homarine	1	9.436
	Caspase-4 activation	8	*A. equina*	0.5 and 0.25	0.235
			A. sulcata	1 and 0.5	15.284
			Homarine	1	42.338

Table S3. *F* values for statistical analysis of PLA_2 assay.

**Assay**	**Extract/Compound**	***F* value**
PLA_2 activity	*A. equina*	18.513
	A. sulcata	4.813
	Homarine	85.570
